# Remote levodopa challenge test in Parkinson's disease: Feasibility, reliability, validity and economic value

**DOI:** 10.1111/ene.16423

**Published:** 2024-08-07

**Authors:** Zhitong Zeng, Zhengyu Lin, Zhonglue Chen, Xiaonan Wan, Haiyan Zhou, Chencheng Zhang, Bomin Sun, Kang Ren, Dianyou Li

**Affiliations:** ^1^ Department of Neurosurgery, Centre for Functional Neurosurgery, Ruijin Hospital Shanghai Jiao Tong University School of Medicine Shanghai China; ^2^ HUST‐GYENNO CNS Intelligent Digital Medicine Technology Centre Wuhan China; ^3^ Department of Neurology and Institute of Neurology, Ruijin Hospital Shanghai Jiao Tong University School of Medicine Shanghai China; ^4^ Clinical Neuroscience Centre, Ruijin Hospital LuWan Branch Shanghai Jiao Tong University School of Medicine Shanghai China

**Keywords:** deep brain stimulation, levodopa challenge test, patients with Parkinson's disease, telemedicine

## Abstract

**Background and purpose:**

The aim was to demonstrate the feasibility, reliability and validity of an in‐home remote levodopa challenge test (LCT), as delivered through an online platform, for patients with Parkinson's disease (PwPD).

**Methods:**

Patients with Parkinson's disease eligible for deep brain stimulation surgery screening were enrolled. Participants sequentially received an in‐home remote LCT and an in‐hospital standard LCT (separated by 2.71 weeks). A modified Movement Disorder Society Unified Parkinson's Disease Rating Scale Part III omitting rigidity and postural stability items was used in the remote LCT. The reliability of the remote LCT was evaluated using the intraclass correlation coefficient and the concurrent validity was evaluated using the Pearson's correlation coefficient *r* between the levodopa responsiveness of the remote and standard LCT.

**Results:**

Out of 106 PwPD screened, 80 (75.5%) completed both the remote and standard LCT. There was a good reliability (intraclass correlation coefficient 0.81, 95% confidence interval 0.69–0.88) and a strong correlation (*r* = 0.84, 95% confidence interval 0.77–0.90) between the levodopa responsiveness of the remote and standard LCT. The mean cost for PwPD was estimated to be reduced by 91% by using the remote LCT.

**Conclusion:**

The remote LCT is feasible, reliable and valid and may reduce healthcare‐related costs for PwPD and their caregivers.

## INTRODUCTION

The gradual and persistent progression of motor and non‐motor impairments in Parkinson's disease (PD) lead to substantial demands and challenges for long‐term healthcare delivery. Symptom assessment and medication adjustment often require regular inpatient visits to the expert centre, which are hindered by high travel costs, lack of available accompaniment and physical immobility. In addition, a higher infection risk in older patients and travel lockdowns during pandemics such as coronavirus disease 2019 have made inpatient visits more challenging. Telemedicine offers a promising solution to enhance patient‐centred medical services and alleviate the burden on patients with PD (PwPD) and their caregivers, which can uniquely provide benefits of ‘the four Cs’ [1], that is, care, convenience, comfort and confidentiality, to PwPD. Specifically, video‐based remote assessment and consultations via smartphones can provide valuable real‐world data that accurately reflect the impact of the disease and non‐medical factors on daily activities and overall quality of life [2].

The levodopa challenge test (LCT), which includes motor evaluation in both off‐ and on‐medication (suprathreshold dose) conditions, is widely used for PD diagnosis and as a screening tool prior to invasive treatments such as deep brain stimulation (DBS), and provides a basis for medication adjustments [3]. Remote LCT via video‐based consultations may help identify the optimal and worst overall motor symptoms in patients in daily life. However, the remote LCT protocol has not been systematically validated. Here, home‐based and in‐hospital LCTs were conducted separately for PD candidates of DBS surgery to investigate the feasibility, reliability and value of remote LCT.

## METHODS

### Participants

From August 2021 to July 2023, patients with PD who were candidates for DBS at Ruijin Hospital were recruited. The inclusion criteria were as follows: (1) diagnosis of idiopathic PD by an experienced movement disorder specialist and (2) disabling motor fluctuations, wearing‐off phenomena or dyskinesia. Exclusion criteria were as follows: (1) decline to participate, (2) not having a 4 × 1.5 m space at home to perform the motor assessment and (3) having dementia. The study protocol was approved by the ethics committee of Ruijin Hospital. All the patients provided written informed consent to participate in this study.

### Assessment platform

The remote LCT assessment was performed on a mobile application named PD CARE (Gyenno Science Co. Ltd), an individualized management platform for PwPD, and the processes were described in detail in the pilot study [4]. Briefly, after the initial consultation, patients who met the inclusion criteria were instructed to use the PD CARE patient portal. A standardized mobile phone holder was sent to the patient and used during the remote assessment. Doctors and assistants performed real‐time online evaluations and collected relevant clinical data via the doctor portal. Online evaluation was videotaped in full. The quality control and measures for data security were described in our previous work [4].

### The LCT protocol

A standard LCT protocol [3] was sequentially conducted at home and in the hospital. Patients withheld all dopaminergic medications for at least 12 h (usually overnight) for the off‐medication condition. Then, a suprathreshold dose of 150% of the patient's morning levodopa equivalent dose was administered, and the state in which the patient reported the peak levodopa efficacy was considered the ‘best’ on‐medication state. A videotaped Movement Disorder Society Unified Parkinson's Disease Rating Scale Part III (MDS UPDRS‐III) motor evaluation was respectively conducted in both off‐ and on‐medication conditions. Notably, a standard MDS UPDRS‐III evaluation was performed for in‐person assessment whilst a modified MDS UPDRS‐III adapted from Abdolahi et al. [5], omitting the items of rigidity (3.3) and postural stability (3.12) with a maximum score of 116, was performed for remote evaluation. Specifically, the participant was required to have a space larger than 4 × 1.5 m at home to perform the motor assessment, especially the timed‐up‐and‐go test needed for the gait, freezing of gait and posture examination. The evaluation time for in‐home and in‐person assessment was controlled to minimize the impact of daily motor fluctuation. The levodopa challenge dose also remained unchanged. Both remote and in‐person evaluations were realized by one certified evaluator. To evaluate the intra‐rater reliability of the modified MDS UPDRS‐III in the remote setting, one‐third of the participants were randomly selected and the same rater re‐scored the videotape 1 year after the initial assessment.

The levodopa response (LR) for motor symptoms, which refers to the relative improvement in the MDS UPDRS‐III score after the LCT, was calculated according to the formula [3]

$$\begin{array}{c} \mathrm{LR}=\frac{“\text{OffMed}”\;\text{score}-\text{peak}\;“\text{OnMed}”\;\text{score}}{“\text{OffMed}”\;\text{score}\;}\times 100\% \end{array}$$

Depending on whether the modified or standard MDS UPDRS‐III was used, the corresponding modified or standard LR was calculated. A series of parkinsonian symptoms were analysed individually: (1) tremor (items 3.15–3.18); (2) bradykinesia (items 3.2, 3.4–3.8 and 3.14); and (3) axial symptoms (items 3.1 and 3.9–3.13).

### Health economic value estimation

The residence address of each participant was recorded to estimate the travel distance, travel cost and travel time for one in‐hospital standard LCT session. The travel cost of a round trip to our centre by high‐speed train or taxi for two individuals (i.e., PwPD and one caregiver) was estimated, whilst an accommodation fee was not included since it was assumed that all patients would return home on the same day. As clinical research, both remote and in‐hospital motor evaluation session were offered free of charge to participants. For the remote assessment, the only cost incurred was the internet fee, estimated at 0.3 renminbi (RMB) per megabyte of data [4].

### Data analysis

The mean value ± standard deviation (SD) or the median value along with range and interquartile range (IQR) was used to describe continuous variables and the frequency for categorical variables, and coefficients were described along with their 95% confidence intervals (CIs). A *p* value <0.05 was considered statistically significant. All statistical analyses were performed using R version 4.0.2 if not specified.

### Validation of the modified MDS UPDRS‐III for remote assessment

Inspired by Abdolahi et al. [5], the reliability of the modified MDS UPDRS‐III (removing rigidity and postural instability items) compared to the standard MDS UPDRS‐III was assessed using intraclass correlation coefficients (ICCs) in both off‐ and on‐medication conditions. An ICC value less than 0.5 indicated poor reliability, between 0.5 and 0.75 indicated moderate reliability, between 0.75 and 0.9 indicated good reliability, and greater than 0.9 indicated excellent reliability [6]. ICC was also used to analyse the intra‐rater reliability of the remote modified MDS UPDRS‐III. Internal consistency of the modified MDS UPDRS‐III was measured using Cronbach's alpha. A value of 0.80 or higher was considered highly consistent. The concurrent validity [7] was calculated by the Pearson's correlation coefficient *r* between scores of remote and standard assessments. *r* values less than 0.4 were considered indicative of a weak correlation, values between 0.4 and 0.7 indicated moderate correlation, values between 0.7 and 0.9 indicated a strong correlation, and values greater than 0.90 indicated a very strong correlation [8]. Of note, the rigidity and postural instability items were excluded from the calculation of ICCs for the total score and axial symptom sub‐score between the modified and standard MDS UPDRS‐III, but were included in the calculation of *r*.

### Validation of the remote LCT

The ICC was calculated to quantify the consistency of the LR between the remote and in‐person standard LCT. The concurrent validity [7] was calculated by Pearson's correlation coefficient *r* between the LR of remote and standard LCTs. Of note, different from the validation of the modified MDS UPDRS‐III, the rigidity and postural instability items were included in the calculation of both ICCs and *r* for the LR on the total score and axial symptom sub‐score between the remote and standard LCTs as the LR was calculated as a ratio. Receiver operating characteristic analysis was further performed to define at which cutoff value and how well the remote LCT could discriminate the partial and full responder in reference to an arbitrary cutoff value of 50% for the standard LCT [9].

### Feasibility and health economic value of the remote LCT

The ratio of the number of PwPD completing remote LCT assessments to the total number of included patients was calculated to assess feasibility [7]. Reasons for not being able to complete the remote assessments were also recorded. To evaluate the health economic value of the remote LCT, a two‐tailed paired *t* test was used to compare the estimated travel distance, cost and time spent between remote and in‐person visits, and a one‐sample chi‐squared test was performed to evaluate patient's preference.

## RESULTS

### Study population

**FIGURE 1:**
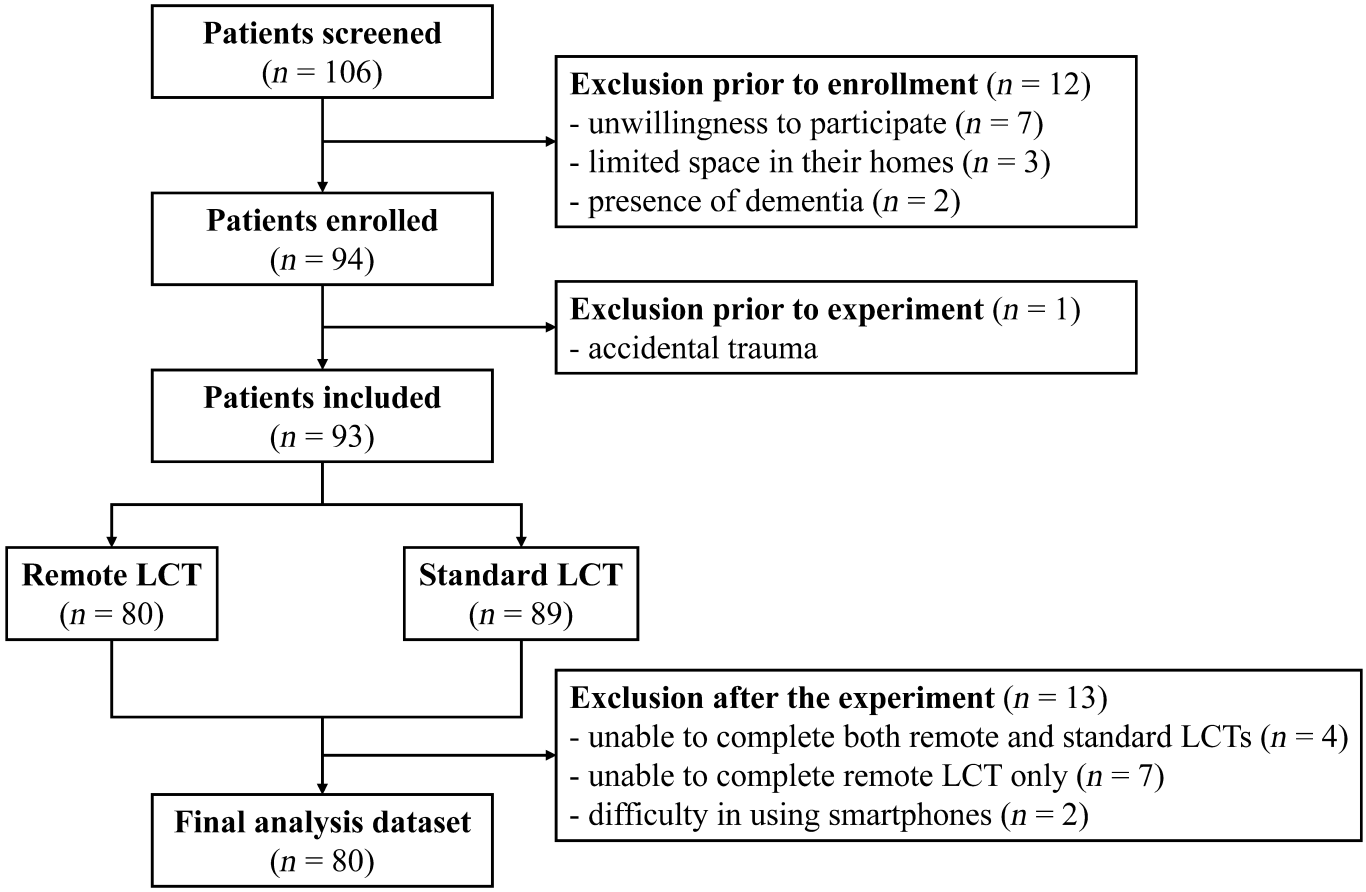
Flowchart detailing the exclusion counts and completed assessments.

Out of 106 PwPD screened, 12 were excluded prior to recruitment due to unwillingness to participate (*n* = 7), limited space in their homes (*n* = 3) and presence of dementia (*n* = 2). One patient was excluded after the enrolment due to accidental trauma. Thus, 93 PwPD sequentially received an in‐home remote LCT and in‐hospital standard LCT. Amongst them, four patients could not tolerate the off‐medication evaluation during both remote and standard LCTs; seven were unable to complete the remote LCT but could complete the standard LCT in the off‐medication state; and two had difficulty in using a smartphone despite detailed instructions during the remote LCT. Therefore, the final analysis dataset consisted of 80 PwPD who completed both in‐home remote and in‐hospital standard LCTs (Figure 1).

The demographical and clinical characteristics are shown in Table 1. Forty‐one participants were females (51.3%, 41/80). The mean age was 64.0 ± 8.0 years, and the mean disease duration of PD was 11.5 ± 4.9 years. The median interval between the in‐home remote LCT and the in‐hospital standard LCT was 2.7 weeks (range 0.1–26 weeks, IQR 1.5–4.9 weeks).

**TABLE 1:** Baseline demographic and clinical characteristics.

Patient characteristics	
Sex, female, *N* (%)	41 (51.3%)
Age (years), mean (range)	64.0 (37–76)
Time of education (years), median (IQR) (range)	10 (8–11) (0–15)
Level of education, *N* (%)	
Middle school or lower	45 (56.25%)
High school	19 (23.75%)
College or higher	16 (20%)
Distance from home to hospital (km), median (IQR) (range)	182.5 (34.25–460) (3.9–2526)
Disease duration (years), mean (range)	11.5 (2–27)
MDS UPDRS‐III in off‐medication state during in‐person LCT, mean (range)	50.0 (14–72)
LR during in‐person LCT, mean (range)	55.7% (26.4%–92.9%)
Time between remote and in‐person LCT (weeks), median (IQR) (range)	2.71 (1.5–4.93) (0.1–26)

Abbreviations: IQR, interquartile range; LR, levodopa response; LCT, levodopa challenge test; MDS UPDRS‐III, Movement Disorder Society Unified Parkinson's Disease Rating Scale Part III.

### Validation of the remote modified MDS UPDRS‐III

The agreement and correlation for each item between the modified and standard MDS UPDRS‐III are shown in Table S1. The ICC (95% CI) between the modified and standard MDS UPDRS‐III total score was 0.89 (0.83–0.93) and 0.92 (0.88–0.95) in the off‐ and on‐medication condition, respectively. With respect to sub‐scores, the ICC (95% CI) was 0.80 (0.70–0.87), 0.73 (0.60–0.82), 0.81 (0.71–0.88) for tremor, bradykinesia and axial symptoms in the off‐medication condition and 0.43 (0.23–0.59), 0.80 (0.71–0.87), 0.59 (0.38–0.74) in the on‐medication condition, respectively (all *p* < 0.001). The concurrent validity, as measured by Pearson's correlation coefficient *r* (95% CI), was 0.88 (0.81–0.92) and 0.89 (0.84–0.93) in the off‐ and on‐medication condition, respectively. With respect to sub‐scores, *r* (95% CI) was 0.81 (0.72–0.87), 0.74 (0.61–0.82), 0.81 (0.72–0.87) for tremor, bradykinesia and axial symptoms in the off‐medication condition, and 0.43 (0.23–0.59), 0.80 (0.71–0.87), 0.66 (0.52–0.77) in the on‐medication condition, respectively (all *p* < 0.001) (Table 2). The internal consistency of the modified MDS UPDRS‐III in the remote setting was high (*α*_remote_ = 0.935, 95% CI 0.920–0.949) and was similar to the standard MDS UPDRS‐III (*α*_standard_ = 0.940, 95% CI 0.926–0.952).

**TABLE 2:** Reliability and concurrent validity of the modified MDS UPDRS‐III for remote assessment.

MDS UPDRS‐III scores	ICC (95% CI)	Level	*p*	*r* (95% CI)	Level	*p*
Off‐medication condition						
Total score^a^	0.89 (0.83–0.93)	Good	<0.001	0.88 (0.81–0.92)	Strong	<0.001
Tremor	0.80 (0.70–0.87)	Good	<0.001	0.81 (0.72–0.87)	Strong	<0.001
Bradykinesia	0.73 (0.60–0.82)	Moderate	<0.001	0.74 (0.61–0.82)	Strong	<0.001
Axial symptoms^b^	0.81 (0.71–0.88)	Good	<0.001	0.81 (0.72–0.87)	Strong	<0.001
On‐medication condition						
Total score^a^	0.92 (0.88–0.95)	Excellent	<0.001	0.89 (0.84–0.93)	Strong	<0.001
Tremor	0.43 (0.23–0.59)	Poor	<0.001	0.43 (0.23–0.59)	Moderate	<0.001
Bradykinesia	0.80 (0.71–0.87)	Good	<0.001	0.80 (0.71–0.87)	Strong	<0.001
Axial symptoms^b^	0.59 (0.38–0.74)	Moderate	<0.001	0.66 (0.52–0.77)	Moderate	<0.001
Levodopa response^c^						
Total score	0.81 (0.69–0.88)	Good	<0.001	0.84 (0.77–0.90)	Strong	<0.001
Tremor	0.38 (0.17–0.56)	Poor	<0.001	0.38 (0.17–0.56)	Weak	<0.001
Bradykinesia	0.63 (0.48–0.75)	Moderate	<0.001	0.63 (0.48–0.75)	Moderate	<0.001
Axial symptoms	0.64 (0.49–0.75)	Moderate	<0.001	0.65 (0.48–0.75)	Moderate	<0.001

*Note*: Classification of reliability levels (ICC): poor (<0.5), moderate (0.5–0.75), good (0.75–0.9), excellent (>0.90). Classification of correlation levels: weak (<0.4), moderate (0.4–0.7), strong (0.7–0.9), very strong (>0.90).

Abbreviations: ICC, intra‐class correlation coefficient; LR, levodopa response; MDS UPDRS‐III, Movement Disorder Society Unified Parkinson's Disease Rating Scale Part III.

^a^ The rigidity and postural instability items were excluded from the calculation of ICCs but included in Pearson's correlation coefficient *r*.

^b^ The postural instability items were excluded from the calculation of ICCs but included in Pearson's correlation coefficient *r*.

^c^ For calculation of levodopa response, the rigidity and postural instability items were included in the calculation of both ICCs and Pearson's correlation coefficient *r*.

With respect to the intra‐rater reliability of the remote modified MDS UPDRS‐III, the ICC (95% CI) was 0.90 (0.762–0.954) and 0.91 (0.818–0.959) in the off‐ and on‐medication condition, respectively (both *p* < 0.001), indicating a good to excellent intra‐rater reliability.

### Validation of the remote LCT

There was a good reliability between the LR on total score of the remote LCT and that of the standard LCT (ICC 0.81, 95% CI 0.69–0.88). Regarding motor sub‐scores, the ICC was 0.38 (0.17–0.56), 0.63 (0.48–0.75) and 0.64 (0.49–0.75) for LR on tremor, bradykinesia and axial symptoms sub‐score between the remote and standard LCT, respectively (all *p* < 0.001). The concurrent validity, as measured by Pearson's correlation coefficient *r* (95% CI) between the LR of the remote LCT and that of the standard LCT was 0.84 (0.77–0.90) on the total score. The correlation between the LR of the remote LCT and that of the standard LCT was weak on tremor (*r* = 0.38, 95% CI 0.17–0.56), moderate on bradykinesia (*r* = 0.63, 95% CI 0.48–0.75) and moderate on axial symptom sub‐score (*r* = 0.65, 95% CI 0.48–0.75) (all *p* < 0.001), respectively (Table 2). The ICC and Pearson's correlation coefficient *r* of the LR on each item between the remote and standard LCT are shown in Table S2.

**FIGURE 2:**
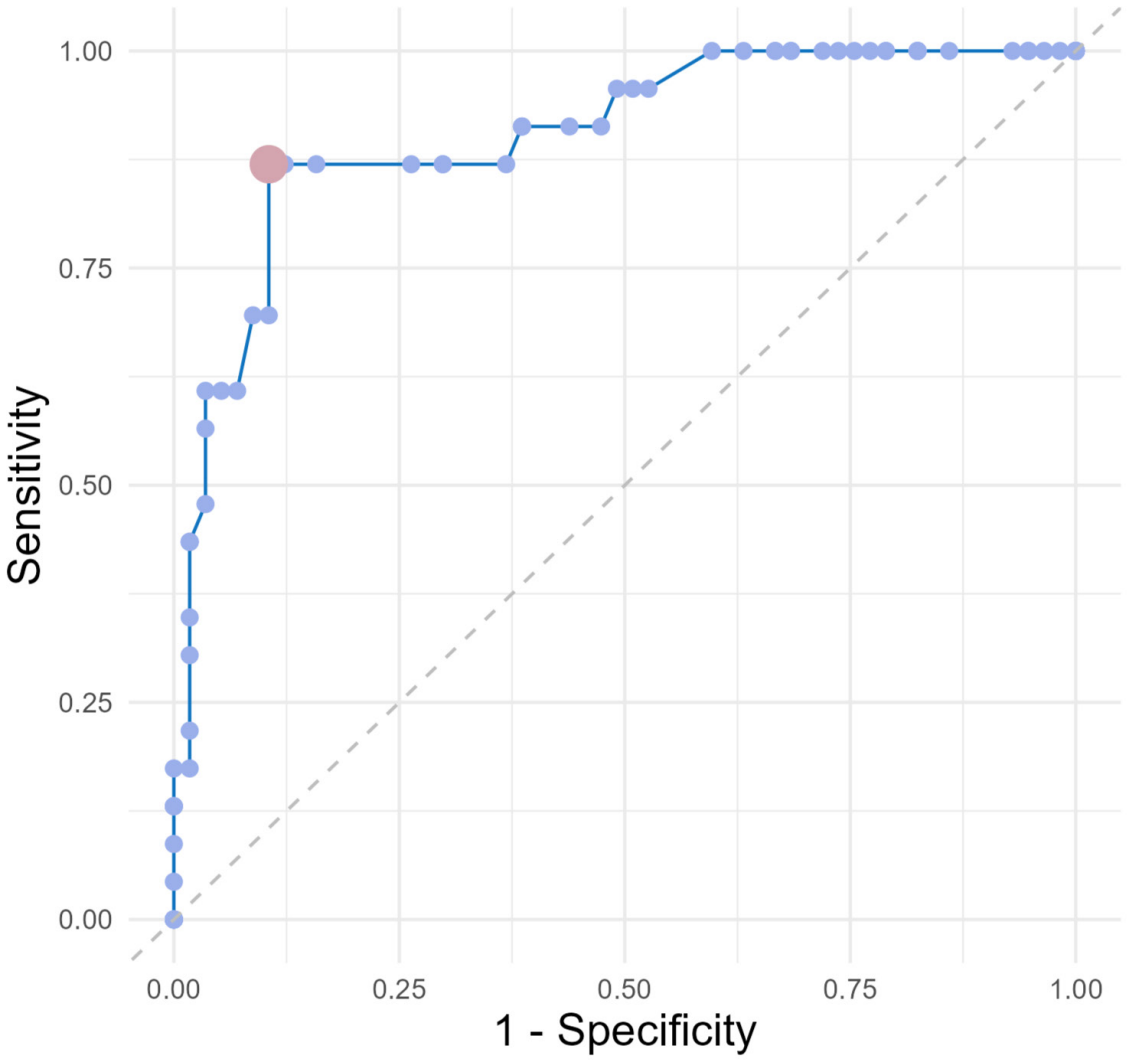
Receiver operating characteristic curve for modified MDS UPDRS‐III in remote assessment. Chosen cutoff point according to the area under the curve is circled.

The ability of the remote LCT to differentiate the partial to full responders of levodopa was further investigated using the LR value of 50% of standard LCT as a reference. Receiver operating characteristic analysis showed that a cutoff value of 45% with an area under the curve of 0.902 (95% CI 0.8258–0.9660, *p* < 0.001) was optimal to distinguish between a partial and full levodopa responder using the remote LCT. The sensitivity was 0.870 and the specificity was 0.895 (Figure 2).

### Feasibility and health economic value of the remote LCT

As mentioned above, 13 out of 93 patients (17.2%) were not able to realize or complete the remote LCT due to intolerance to the off‐medication condition (*n* = 11) and difficulty in using the platform on the smartphone (*n* = 2). No adverse events were documented during the virtual evaluation.

Participants resided in 13 provinces in China, and the median distance from their residence to our centre was 182.5 km (range 3.9–2526 km, IQR 34.25–460 km). For an in‐home remote LCT session, the mean estimated cost, summing the travel, evaluation and network costs, was 96% (21.0 vs. 469.5 RMB, *p* < 0.001) less than that for an in‐hospital standard session. The time spent for one remote LCT session was significantly longer than that required for a standard session (remote 41.5 ± 14.2 min, standard 21.4 ± 6.7 min; *p* < 0.001), whilst the total time required including the travel time was reduced by 91% for a remote LCT session compared to the standard session (remote 0.69 ± 0.24 h, standard 7.66 ± 4.47 h, *p* < 0.001).

## DISCUSSION

In this study, it was demonstrated that a remote LCT, along with a modified MDS UPDRS‐III, was a reliable and valid tool for eligibility screening prior to DBS surgery for PwPD. It was feasible to be conducted in the homes of most participants and would alleviate the healthcare‐related burdens of PwPD in terms of reducing the travel cost and time to the expert centre.

A moderate agreement between remote and in‐person standard motor assessment has been reported by several groups [7, 10, 11]. Indeed, environmental (e.g., lighting, background and space), participant (e.g., age, access/familiarity with technology, disease stage and cognitive status) and investigator factors (e.g., experience in remote assessment and inter‐rater reliability) are main challenges that may affect the accuracy of the remote assessment [10]. Therefore, an attempt was made to minimize these impacts by controlling the interval between remote and in‐person session, the time point of the assessment, the levodopa challenge dose and the experienced assessor.

Although the overall reliability of the remote LCT was high, the level of consistency for specific motor symptoms was different. For example, the agreement of the tremor sub‐score between the remote and in‐person assessment was the lowest compared to sub‐scores of bradykinesia and axial symptoms in both off‐ and on‐medication conditions. This is in line with the current literature that the tremor could be underrated during the remote assessment as subtle tremor can be difficult to appreciate remotely due to several factors including the camera distance, video resolution and quality of network connection [10, 12]. Moreover, the consistency of MDS UPDRS‐III scores in the on‐medication condition was lower than that in the off‐medication condition, probably due to more subtle symptoms that were more difficult to be discerned remotely [7, 11]. These issues might be addressed by integrating more advanced technologies into the telemedicine platform such as computer vision, deep vision and wearable sensors.

The remote LCT was demonstrated to reduce the healthcare‐related travel cost and time. Consistently, the feasibility and healthcare economic value of telemedicine in PD have been previously reported [4, 10, 13]. Many PwPD do not have adequate access to healthcare services, including but not limited to a regular monitoring of motor and non‐motor impairments, medication adjustment and surgery consultation, due to geographical distance to an expert centre, limited local medical services, motor disability, unavailability of caregivers, and so on. Fortunately, PD telemedicine via an online platform would fill the gap to make these services more accessible. The demand for such amenities is particularly strong in China, with its large patient base, vast geographical area and relatively limited medical resources [4].

Several caveats should be underlined in remote LCT based on our experience. First, a stable and fast internet connection is fundamental for a reliable remote motor assessment as a significant delay and/or video stuttering may considerably alter the rating of the motor performance, for example the tremor and bradykinesia. Secondly, at least one caregiver should be present during the remote assessment to ensure the security of the patient (e.g., falls, balance issue).

This study also has several limitations. In the first place, the disease stage of the enrolled PwPD was relatively advanced as they were candidates for DBS surgery. Consequently, motor impairments could be more easily and accurately evaluated during remote assessments compared to those in earlier stages. Secondly, the inter‐rater reliability of the remote LCT was not assessed in the present study. Finally, the feasibility of remote PD motor assessment may, at least in part, depend on the accessibility of the online platform, and the generalizability of our findings to other platforms remains to be explored.

## AUTHOR CONTRIBUTIONS

**Zhitong Zeng:** Investigation; validation; writing – original draft; conceptualization; data curation; formal analysis; methodology. **Zhengyu Lin:** Data curation; writing – review and editing. **Zhonglue Chen:** Data curation; validation; writing – review and editing; visualization; formal analysis. **Xiaonan Wan:** Data curation; validation. **Haiyan Zhou:** Validation; data curation. **Chencheng Zhang:** Validation; data curation; writing – review and editing. **Bomin Sun:** Conceptualization; supervision; validation; writing – review and editing. **Kang Ren:** Methodology; validation; visualization. **Dianyou Li:** Conceptualization; methodology; validation; supervision; project administration; funding acquisition; writing – review and editing; resources.

## FUNDING INFORMATION

This study was funded by the Shanghai Municipal Health Commission (202,140,181 to DYL), the Shanghai Science and Technology Commission (22Y11903900 to DYL) and Guangci Innovative Technology Launch Programme (GCQH202205) to DYL.

## CONFLICT OF INTEREST STATEMENT

Z.C. and K.R. were employed by the company Gyenno Science Co. Ltd, Shenzhen, China. No authors have any conflict of interest to declare.

## Supporting information


Table S1.

## Data Availability

The dataset generated during the present study is available from the corresponding author upon reasonable request.

## References

[1] Dorsey ER , Okun MS , Bloem BR . Care, convenience, comfort, confidentiality, and contagion: the 5 C's that will shape the future of telemedicine. J Parkinsons Dis. 2020;10(3):893‐897. doi:10.3233/JPD-202109 32538870

[2] Omberg L , Chaibub Neto E , Perumal TM , et al. Remote smartphone monitoring of Parkinson's disease and individual response to therapy. Nat Biotechnol. 2022;40(4):480‐487. doi:10.1038/s41587-021-00974-9 34373643 PMC12812035

[3] Saranza G , Lang AE . Levodopa challenge test: indications, protocol, and guide. J Neurol. 2020;268:3135‐3143. doi:10.1007/s00415-020-09810-7 32333167

[4] Xu X , Zeng Z , Qi Y , et al. Remote video‐based outcome measures of patients with Parkinson's disease after deep brain stimulation using smartphones: a pilot study. Neurosurg Focus. 2021;51(5):E2. doi:10.3171/2021.8.FOCUS21383 34724646

[5] Abdolahi A , Scoglio N , Killoran A , Dorsey ER , Biglan KM . Potential reliability and validity of a modified version of the unified Parkinson's disease rating scale that could be administered remotely. Parkinsonism Relat Disord. 2013;19(2):218‐221. doi:10.1016/j.parkreldis.2012.10.008 23102808 PMC3666325

[6] Koo TK , Li MY . A guideline of selecting and reporting intraclass correlation coefficients for reliability research. J Chiropr Med. 2016;15(2):155‐163. doi:10.1016/j.jcm.2016.02.012 27330520 PMC4913118

[7] Cubo E , Trejo Gabriel‐Galán JM , Seco Martínez J , et al. Comparison of office‐based versus home web‐based clinical assessments for Parkinson's disease. Mov Disord. 2012;27(2):308‐311. doi:10.1002/mds.24028 22173694

[8] Schober P , Boer C , Schwarte LA . Correlation coefficients: appropriate use and interpretation. Anesth Analg. 2018;126(5):1763‐1768. doi:10.1213/ANE.0000000000002864 29481436

[9] Deuschl G , Follett KA , Luo P , et al. Comparing two randomized deep brain stimulation trials for Parkinson's disease. J Neurosurg. 2020;132(5):1376‐1384. doi:10.3171/2018.12.JNS182042 30952118

[10] Schneider RB , Myers TL , Tarolli CG , et al. Remote administration of the MDS‐UPDRS in the time of COVID‐19 and beyond. J Parkinsons Dis. 2020;10(4):1379‐1382. doi:10.3233/JPD-202121 32675421 PMC12803732

[11] Tarolli CG , Andrzejewski K , Zimmerman GA , et al. Feasibility, reliability, and value of remote video‐based trial visits in Parkinson's disease. J Parkinsons Dis. 2020;10(4):1779‐1786. doi:10.3233/JPD-202163 32894251

[12] Stillerova T , Liddle J , Gustafsson L , Lamont R , Silburn P . Remotely assessing symptoms of Parkinson's disease using videoconferencing: a feasibility study. Neurol Res Int. 2016;2016:4802570. doi:10.1155/2016/4802570 28116158 PMC5220499

[13] Larson DN , Schneider RB , Simuni T . A new era: the growth of video‐based visits for remote management of persons with Parkinson's disease. J Parkinsons Dis. 2021;11(s1):S27‐S34. doi:10.3233/JPD-202381 33492246 PMC8385503

