# Supplementary material for: Remote levodopa challenge test in Parkinson's disease: Feasibility, reliability, validity and economic value
Source: Eur J Neurol. 2024 Aug 7;31(10):e16423. doi: 10.1111/ene.16423 (PMC11414795; doi:10.1111/ene.16423)
Supplement: Supplementary file 1 — Table S1. [file ENE-31-e16423-s001.docx]

**Supplementary table 1. Agreement and correlation of each item in modified MDS UPDRS-III between in-person and remote assessments.**

| **Item in MDS-UPRDS Part Ⅲ** | **Off-medication** | | | | | | **On-medication** | | | | | |
| --- | --- | --- | --- | --- | --- | --- | --- | --- | --- | --- | --- | --- |
|  | **Remote LCT** | **In-person LCT** | **ICC (95%CI)** | ***p*** | ***r* (95%CI)** | ***p*** | **Remote LCT** | **In-person LCT** | **ICC (95%CI)** | ***p*** | ***r* (95%CI)** | ***p*** |
| 3.1 Speech | 1.05 ± 0.83 | 0.72 ± 0.62 | 0.33 (0.12–0.51) | 0.001 | 0.37 (0.17–0.55) | <0.001 | 0.72 ± 0.71 | 0.51 ± 0.6 | 0.4 (0.2–0.57) | <0.001 | 0.37 (0.17–0.55) | <0.001 |
| 3.2 Facial Expression | 2.52 ± 1.14 | 2.76 ± 1.07 | 0.6 (0.44–0.73) | <0.001 | 0.57 (0.40–0.70) | <0.001 | 1.99 ± 1.06 | 2.29 ± 1.13 | 0.48 (0.3–0.64) | <0.001 | 0.46 (0.27–0.62) | <0.001 |
| 3.4 Finger Tapping | 1.69 ± 0.88 | 1.68 ± 0.73 | 0.55 (0.43–0.65) | <0.001 | 0.56 (0.44–0.65) | <0.001 | 0.96 ± 0.8 | 0.96 ± 0.77 | 0.59 (0.48–0.68) | <0.001 | 0.59 (0.48–0.68) | <0.001 |
| 3.5 Hand Movements | 1.3 ± 0.84 | 1.42 ± 0.73 | 0.43 (0.3–0.55) | <0.001 | 0.44 (0.31–0.56) | <0.001 | 0.64 ± 0.71 | 0.81 ± 0.76 | 0.49 (0.36–0.6) | <0.001 | 0.50 (0.38–0.61) | <0.001 |
| 3.6 Pronation-Supination Movements of Hands | 1.71 ± 0.78 | 1.79 ± 0.72 | 0.54 (0.42–0.64) | <0.001 | 0.55 (0.43–0.65) | <0.001 | 1.04 ± 0.74 | 0.96 ± 0.77 | 0.55 (0.43–0.65) | <0.001 | 0.55 (0.44–0.65) | <0.001 |
| 3.7 Toe Tapping | 1.93 ± 1.06 | 1.83 ± 0.88 | 0.54 (0.42–0.64) | <0.001 | 0.55 (0.43–0.65) | <0.001 | 1.09 ± 0.93 | 1.09 ± 0.76 | 0.5 (0.37–0.6) | <0.001 | 0.51 (0.38–0.61) | <0.001 |
| 3.8 Leg Agility | 1.52 ± 1.05 | 1.41 ± 0.98 | 0.63 (0.53–0.72) | <0.001 | 0.60 (0.49–0.69) | <0.001 | 0.66 ± 0.74 | 0.44 ± 0.68 | 0.43 (0.29–0.55) | <0.001 | 0.46 (0.33–0.57) | <0.001 |
| 3.9 Arising from Chair | 0.96 ± 1.04 | 0.92 ± 1.11 | 0.79 (0.69–0.86) | <0.001 | 0.62 (0.46–0.74) | <0.001 | 0.31 ± 0.65 | 0.22 ± 0.53 | 0.38 (0.17–0.55) | <0.001 | 0.41 (0.21–0.58) | <0.001 |
| 3.10. Gait | 2.04 ± 0.92 | 2.09 ± 0.77 | 0.69 (0.55–0.79) | <0.001 | 0.52 (0.34–0.66) | <0.001 | 1.12 ± 0.83 | 0.9 ± 0.72 | 0.46 (0.27–0.62) | <0.001 | 0.48 (0.29–0.63) | <0.001 |
| 3.11. Freezing Of Gait | 1.29 ± 1.49 | 1.27 ± 1.5 | 0.77 (0.66–0.84) | <0.001 | 0.82 (0.73–0.88) | <0.001 | 0.3 ± 0.8 | 0.2 ± 0.66 | 0.63 (0.48–0.75) | <0.001 | 0.56 (0.39–0.69) | <0.001 |
| 3.13 Posture | 1.65 ± 1.02 | 1.34 ± 1.08 | 0.72 (0.56–0.82) | <0.001 | 0.75 (0.64–0.84) | <0.001 | 0.92 ± 1.05 | 0.66 ± 0.89 | 0.7 (0.55–0.8) | <0.001 | 0.69 (0.56–0.79) | <0.001 |
| 3.14 Global Spontaneity of Movement (Body Bradykinesia) | 1.98 ± 0.87 | 1.96 ± 0.63 | 0.49 (0.3–0.64) | <0.001 | 0.53 (0.35–0.67) | <0.001 | 0.79 ± 0.67 | 0.82 ± 0.65 | 0.58 (0.42–0.71) | <0.001 | 0.56 (0.39–0.69) | <0.001 |
| 3.16 Postural Tremor | 0.82 ± 0.94 | 1 ± 0.66 | 0.58 (0.47–0.68) | <0.001 | 0.50 (0.37–0.61) | <0.001 | 0.36 ± 0.68 | 0.65 ± 0.53 | 0.34 (0.18–0.49) | <0.001 | 0.38 (0.23–0.50) | <0.001 |
| 3.16. Kinetic Tremor | 0.81 ± 0.73 | 0.65 ± 0.64 | 0.5 (0.37–0.61) | <0.001 | 0.48 (0.35–0.59) | <0.001 | 0.43 ± 0.56 | 0.4 ± 0.54 | 0.45 (0.32–0.56) | <0.001 | 0.49 (0.36–0.60) | <0.001 |
| 3.17. Rest Tremor of Lip/Jaw | 0.11 ± 0.42 | 0.24 ± 0.58 | 0.69 (0.59–0.76) | <0.001 | 0.42 (0.22–0.59) | <0.001 | 0.01 ± 0.11 | 0.03 ± 0.16 | 0.39 (0.25–0.51) | <0.001 | -0.02 (-0.24–0.20) | 0.874 |
| 3.17. Rest Tremor Upper Extremities | 0.67 ± 1.06 | 0.65 ± 0.95 | 0.52 (0.34–0.67) | <0.001 | 0.71 (0.62–0.78) | <0.001 | 0.09 ± 0.38 | 0.05 ± 0.31 | 0* | 0.560 | 0.13 (-0.03–0.28) | 0.104 |
| 3.17. Rest Tremor of Lower Extremities | 0.68 ± 0.88 | 0.84 ± 0.94 | 0.79 (0.72–0.84) | <0.001 | 0.68 (0.59–0.75) | <0.001 | 0.09 ± 0.3 | 0.04 ± 0.28 | 0.07 (-0.09–0.22) | 0.196 | 0.39 (0.25–0.52) | <0.001 |
| 3.18. Rest Constancy | 1.86 ± 1.69 | 2.35 ± 1.81 | 0.7 (0.54–0.8) | <0.001 | 0.70 (0.57–0.80) | <0.001 | 0.25 ± 0.61 | 0.2 ± 0.74 | 0.36 (0.16–0.54) | <0.001 | 0.27 (0.06–0.46) | 0.014 |

Scores of items were shown by Mean ± SD.

Abbreviations: MDS-UPDRS Part Ⅲ, Movement Disorder Society Unified Parkinson’s Disease Rating Scale Part Ⅲ; ICC, intraclass correlation coefficient; 95%CI, 95% confidence intervals.

* resetted the ICC to 0 because of the negative value (J J Bartko, Psychological Bulletin, 1976)

**Supplementary table 2. Agreement and correlation of levodopa response of each item between in-person and remote assessments.**

| **Item in MDS-UPRDS Part Ⅲ** | **Levodopa response** | | | | | | |
| --- | --- | --- | --- | --- | --- | --- | --- |
|  | **Sample size*** | **Remote LCT** | **In-person LCT** | **ICC (95%CI)** | ***p*** | **r (95%CI)** | ***p*** |
| 3.1 Speech | 43 | 0.33 ± 0.41 | 0.3 ± 0.45 | 0.49 (0.22–0.69) | <0.001 | 0.46 (0.18–0.67) | 0.002 |
| 3.2 Facial Expression | 75 | 0.22 ± 0.34 | 0.18 ± 0.28 | 0.35 (0.14–0.53) | <0.001 | 0.28 (0.06–0.48) | 0.014 |
| 3.4 Finger Tapping | 144 | 0.44 ± 0.41 | 0.46 ± 0.44 | 0.35 (0.20–0.49) | <0.001 | 0.37 (0.22–0.51) | <0.001 |
| 3.5 Hand Movements | 130 | 0.5 ± 0.49 | 0.46 ± 0.44 | 0.29 (0.13–0.44) | <0.001 | 0.29 (0.13–0.44) | <0.001 |
| 3.6 Pronation-Supination Movements of Hands | 148 | 0.41 ± 0.4 | 0.48 ± 0.41 | 0.37 (0.23–0.50) | <0.001 | 0.35 (0.2–0.49) | <0.001 |
| 3.7 Toe Tapping | 145 | 0.46 ± 0.4 | 0.39 ± 0.4 | 0.13 (-0.03–0.28) | 0.057548 | 0.19 (0.03–0.35) | 0.019 |
| 3.8 Leg Agility | 118 | 0.58 ± 0.43 | 0.73 ± 0.38 | 0.30 (0.12–0.45) | 0.00048 | 0.32 (0.15–0.48) | <0.001 |
| 3.9 Arising from Chair | 37 | 0.68 ± 0.44 | 0.8 ± 0.34 | 0.41 (0.10–0.64) | 0.005302 | 0.38 (0.06–0.62) | 0.021 |
| 3.10. Gait | 78 | 0.46 ± 0.37 | 0.58 ± 0.33 | 0.34 (0.13–0.52) | <0.001 | 0.36 (0.14–0.54) | 0.001 |
| 3.11. Freezing Of Gait | 37 | 0.74 ± 0.42 | 0.83 ± 0.37 | 0.49 (0.20–0.70) | <0.001 | 0.49 (0.2–0.7) | 0.002 |
| 3.13 Posture | 57 | 0.45 ± 0.47 | 0.58 ± 0.41 | 0.52 (0.30–0.69) | <0.001 | 0.55 (0.34–0.71) | <0.001 |
| 3.14 Global Spontaneity of Movement (Body Bradykinesia) | 76 | 0.6 ± 0.32 | 0.6 ± 0.32 | 0.37 (0.16–0.55) | <0.001 | 0.45 (0.24–0.61) | <0.001 |
| 3.16 Postural Tremor | 83 | 0.59 ± 0.48 | 0.33 ± 0.44 | 0.18 (-0.02–0.37) | 0.036941 | 0.21 (0–0.41) | 0.052 |
| 3.16. Kinetic Tremor | 75 | 0.48 ± 0.51 | 0.47 ± 0.5 | 0.25 (0.02–0.45) | 0.015361 | 0.32 (0.11–0.51) | 0.004 |
| 3.17. Rest Tremor of Lip/Jaw | 4 | 0.92 ± 0.2 | 0.85 ± 0.38 | 0 | 0.5 | NA | NA |
| 3.17. Rest Tremor Upper Extremities | 43 | 0.88 ± 0.29 | 0.95 ± 0.21 | 0.02 (-0.28–0.32) | 0.451175 | 0.04 (-0.26–0.34) | 0.790 |
| 3.17. Rest Tremor of Lower Extremities | 56 | 0.87 ± 0.29 | 0.96 ± 0.19 | 0.46 (0.22–0.64) | 0.000163 | 0.47 (0.23–0.65) | <0.001 |
| 3.18. Rest Constancy | 47 | 0.88 ± 0.23 | 0.92 ± 0.22 | 0.29 (0.00–0.53) | 0.024498 | 0.23 (-0.06–0.48) | 0.125 |

Scores of items were shown by Mean ± SD.

Abbreviations: MDS-UPDRS Part Ⅲ, Movement Disorder Society Unified Parkinson’s Disease Rating Scale Part Ⅲ; ICC, intraclass correlation coefficient; 95%CI, 95% confidence intervals.

*Items related to both sides of the body was treated as two separate samples. If the score in the off-medication condition was zero in either remote or in-person assessment, the LR could not be calculated and that particular sample would be excluded.

**Supplementary table 3-1. The p-values of one-tailed t-test between** **MAE results of prediction models using different numbers of items^*^.**

| **Number of items** | **2** | **3** | **4** | **5** | **6** | **7** | **8** | **9** | **10** | **11** | **12** | **13** | **14** | **15** | **16** | **17** | **18** |
| --- | --- | --- | --- | --- | --- | --- | --- | --- | --- | --- | --- | --- | --- | --- | --- | --- | --- |
| **2** | 0.500 | 0.900 | 0.999 | 1.000 | 1.000 | 1.000 | 1.000 | 1.000 | 1.000 | 1.000 | 1.000 | 1.000 | 1.000 | 1.000 | 1.000 | 1.000 | 1.000 |
| **3** | 0.100 | 0.500 | 0.973 | 0.999 | 1.000 | 1.000 | 1.000 | 1.000 | 1.000 | 1.000 | 1.000 | 1.000 | 1.000 | 1.000 | 1.000 | 1.000 | 1.000 |
| **4** | 0.001 | 0.027 | 0.500 | 0.846 | 0.917 | 0.989 | 0.996 | 0.998 | 0.997 | 0.999 | 0.998 | 0.998 | 0.998 | 0.997 | 0.997 | 0.996 | 0.996 |
| **5** | <0.001 | 0.001 | 0.154 | 0.500 | 0.647 | 0.916 | 0.962 | 0.975 | 0.969 | 0.985 | 0.981 | 0.980 | 0.975 | 0.971 | 0.971 | 0.966 | 0.960 |
| **6** | <0.001 | <0.001 | 0.083 | 0.353 | 0.500 | 0.847 | 0.924 | 0.947 | 0.936 | 0.966 | 0.959 | 0.958 | 0.948 | 0.940 | 0.939 | 0.930 | 0.919 |
| **7** | <0.001 | <0.001 | 0.011 | 0.084 | 0.153 | 0.500 | 0.659 | 0.725 | 0.685 | 0.785 | 0.761 | 0.756 | 0.723 | 0.700 | 0.697 | 0.674 | 0.646 |
| **8** | <0.001 | <0.001 | 0.004 | 0.038 | 0.076 | 0.341 | 0.500 | 0.575 | 0.525 | 0.645 | 0.616 | 0.610 | 0.571 | 0.545 | 0.540 | 0.515 | 0.485 |
| **9** | <0.001 | <0.001 | 0.002 | 0.025 | 0.053 | 0.275 | 0.425 | 0.500 | 0.449 | 0.572 | 0.541 | 0.536 | 0.495 | 0.470 | 0.464 | 0.440 | 0.410 |
| **10** | <0.001 | <0.001 | 0.003 | 0.031 | 0.064 | 0.315 | 0.475 | 0.551 | 0.500 | 0.624 | 0.593 | 0.588 | 0.547 | 0.521 | 0.516 | 0.490 | 0.459 |
| **11** | <0.001 | <0.001 | 0.001 | 0.015 | 0.034 | 0.215 | 0.355 | 0.428 | 0.376 | 0.500 | 0.469 | 0.463 | 0.422 | 0.398 | 0.392 | 0.368 | 0.340 |
| **12** | <0.001 | <0.001 | 0.002 | 0.019 | 0.041 | 0.239 | 0.384 | 0.459 | 0.407 | 0.531 | 0.500 | 0.495 | 0.453 | 0.428 | 0.423 | 0.398 | 0.369 |
| **13** | <0.001 | <0.001 | 0.002 | 0.020 | 0.042 | 0.244 | 0.390 | 0.464 | 0.412 | 0.537 | 0.505 | 0.500 | 0.459 | 0.434 | 0.428 | 0.403 | 0.374 |
| **14** | <0.001 | <0.001 | 0.002 | 0.025 | 0.052 | 0.277 | 0.429 | 0.505 | 0.453 | 0.578 | 0.547 | 0.541 | 0.500 | 0.474 | 0.469 | 0.444 | 0.414 |
| **15** | <0.001 | <0.001 | 0.003 | 0.029 | 0.060 | 0.300 | 0.455 | 0.530 | 0.479 | 0.602 | 0.572 | 0.566 | 0.526 | 0.500 | 0.495 | 0.470 | 0.439 |
| **16** | <0.001 | <0.001 | 0.003 | 0.029 | 0.061 | 0.303 | 0.460 | 0.536 | 0.484 | 0.608 | 0.577 | 0.572 | 0.531 | 0.505 | 0.500 | 0.474 | 0.444 |
| **17** | <0.001 | <0.001 | 0.004 | 0.034 | 0.070 | 0.326 | 0.485 | 0.560 | 0.510 | 0.632 | 0.602 | 0.597 | 0.556 | 0.530 | 0.526 | 0.500 | 0.470 |
| **18** | <0.001 | <0.001 | 0.004 | 0.040 | 0.081 | 0.354 | 0.515 | 0.590 | 0.541 | 0.660 | 0.631 | 0.626 | 0.586 | 0.561 | 0.556 | 0.530 | 0.500 |

Blue color indicated the p-values <0.05, and green color represented the number of items selected accordingly.

* Input: items of remote MDS-UDPRS Part Ⅲ without 3.14 (global spontaneity of movement) and 3.18 (constancy of rest tremor), with r>=0.4 and p value of r <0.05; output: total score of in-person MDS-UPSRS Part Ⅲ (including 3.3 rigidity and 3.12 posture stability).

Abbreviations: MAE, mean absolute error; MDS-UPDRS Part Ⅲ, Movement Disorder Society Unified Parkinson’s Disease Rating Scale Part Ⅲ.

**Supplementary table 3-2. Parameters of prediction models using different numbers of items.**

| **Number of items** | **MAE** | ***r* (95%CI)** | ***p* value of *r*** | **ICC (95%CI)** | ***p* value of ICC** |
| --- | --- | --- | --- | --- | --- |
| **2** | 9.225 | 0.772 (0.701–0.828) | <0.001 | 0.750 (0.673–0.811) | <0.001 |
| **3** | 8.263 | 0.816 (0.757–0.862) | <0.001 | 0.802 (0.739–0.851) | <0.001 |
| **4** | 6.913 | 0.864 (0.818–0.899) | <0.001 | 0.856 (0.809–0.893) | <0.001 |
| **5** | 6.281 | 0.897 (0.862–0.924) | <0.001 | 0.893 (0.856–0.920) | <0.001 |
| **6** | 6.075 | 0.906 (0.873–0.930) | <0.001 | 0.902 (0.869–0.928) | <0.001 |
| **7** | 5.544 | 0.919 (0.891–0.940) | <0.001 | 0.917 (0.888–0.938) | <0.001 |
| **8** | 5.338 | 0.924 (0.897–0.944) | <0.001 | 0.922 (0.895–0.942) | <0.001 |
| **9** | 5.244 | 0.926 (0.900–0.945) | <0.001 | 0.924 (0.898–0.944) | <0.001 |
| **10** | 5.306 | 0.927 (0.901–0.946) | <0.001 | 0.925 (0.899–0.945) | <0.001 |
| **11** | 5.156 | 0.930 (0.906–0.949) | <0.001 | 0.929 (0.904–0.947) | <0.001 |
| **12** | 5.194 | 0.929 (0.904–0.948) | <0.001 | 0.928 (0.903–0.947) | <0.001 |
| **13** | 5.200 | 0.929 (0.904–0.947) | <0.001 | 0.927 (0.902–0.946) | <0.001 |
| **14** | 5.250 | 0.928 (0.902–0.946) | <0.001 | 0.927 (0.901–0.946) | <0.001 |
| **15** | 5.281 | 0.926 (0.900–0.945) | <0.001 | 0.925 (0.899–0.945) | <0.001 |
| **16** | 5.288 | 0.927 (0.901–0.946) | <0.001 | 0.926 (0.900–0.945) | <0.001 |
| **17** | 5.319 | 0.926 (0.900–0.945) | <0.001 | 0.925 (0.899–0.944) | <0.001 |
| **18** | 5.356 | 0.925 (0.898–0.944) | <0.001 | 0.924 (0.897–0.944) | <0.001 |

Green color represented the number of items selected.

Abbreviations: MAE, mean absolute error; ICC, intraclass correlation coefficient; *r*, Pearson correlation coefficient; 95%CI, 95% confidence intervals.

**Supplementary table 3-3. Ranking of items by beta-value in the prediction models.**

| **Rank** | **Item** | **Name of item** | **Beta** | ***p* value of beta** | ***r* (95%CI)** | ***p* value of *r*** |
| --- | --- | --- | --- | --- | --- | --- |
| 1 | 3.6 | Pronation-Supination Movements of Hands._mean | 3.350 | <0.001 | 0.664(0.567–0.743) | <0.001 |
| 2 | 3.10 | Gait | 3.152 | <0.001 | 0.617(0.511–0.705) | <0.001 |
| 3 | 3.13 | Posture | 2.912 | <0.001 | 0.558(0.442–0.657) | <0.001 |
| 4 | 3.17 | Rest Tremor LE._mean | 2.835 | <0.001 | 0.480(0.351–0.591) | <0.001 |
| 5 | 3.17 | Rest Tremor UE._max | 2.825 | <0.001 | 0.528(0.406–0.631) | <0.001 |
| 6 | 3.16 | Kinetic Tremor._min | 2.565 | <0.001 | 0.472(0.342–0.585) | <0.001 |
| 7 | 3.7 | Toe Tapping._mean | 2.243 | <0.001 | 0.649(0.587–0.756) | <0.001 |
| 8 | 3.4 | Finger Tapping._mean | 2.228 | <0.001 | 0.680(0.549–0.73) | <0.001 |
| 9 | 3.9 | Arising From Chair | 1.767 | <0.001 | 0.593(0.482–0.685) | <0.001 |
| 10 | 3.1 | Speech | 1.445 | <0.001 | 0.415(0.278–0.536) | <0.001 |
| 11 | 3.16 | Postural Tremor._min | 1.440 | <0.001 | 0.471(0.341–0.584) | <0.001 |
| 12 | 3.17 | Rest Tremor LE._min | 1.356 | <0.001 | 0.431(0.296–0.55) | <0.001 |
| 13 | 3.6 | Pronation-Supination Movements of Hands._max | 1.334 | <0.001 | 0.635(0.532–0.719) | <0.001 |
| 14 | 3.8 | Leg Agility._max | 1.034 | <0.001 | 0.668(0.572–0.746) | <0.001 |
| 15 | 3.17 | Rest Tremor UE._mean | 0.806 | <0.001 | 0.571(0.457–0.667) | <0.001 |
| 16 | 3.16 | Postural Tremor._mean | 0.597 | <0.001 | 0.520(0.397–0.625) | <0.001 |
| 17 | 3.5 | Hand Movements._max | 0.487 | <0.001 | 0.630(0.527–0.715) | <0.001 |
| 18 | 3.4 | Finger Tapping._min | 0.002 | <0.001 | 0.653(0.554–0.734) | <0.001 |

Green color represented the items selected.

Abbreviations: MAE, mean absolute error; *r*, Pearson correlation coefficient; LE, lower extremities; UE, upper extremities.
